# Hydrogen Sulfide Offers Neuroprotection on Traumatic Brain Injury in Parallel with Reduced Apoptosis and Autophagy in Mice

**DOI:** 10.1371/journal.pone.0087241

**Published:** 2014-01-23

**Authors:** Mingyang Zhang, Haiyan Shan, Pan Chang, Tao Wang, Wenwen Dong, Xiping Chen, Luyang Tao

**Affiliations:** 1 Department of Forensic Science and Laboratory of Brain Injury, Medical College of Soochow University, Suzhou, China; 2 Department of Forensic Science, Medical College of Nantong University, Nantong, China; 3 Department of Obstetrics and Gynecology, North District of Suzhou Municipal Hospital, Suzhou, China; University of Louisville, United States of America

## Abstract

Hydrogen sulfide (H_2_S), a novel gaseous mediator, has been recognized as an important neuromodulator and neuroprotective agent in the central nervous system. The present study was undertaken to study the effects of exogenous H_2_S on traumatic brain injury (TBI) and the underlying mechanisms. The effects of exogenous H_2_S on TBI were examined by using measurement of brain edema, behavior assessment, propidium iodide (PI) staining, and Western blotting, respectively. Compared to TBI groups, H_2_S pretreatment had reduced brain edema, improved motor performance and ameliorated performance in Morris water maze test after TBI. Immunoblotting results showed that H_2_S pretreatment reversed TBI-induced cleavage of caspase-3 and decline of Bcl-2, suppressed LC3-II, Beclin-1 and Vps34 activation and maintained p62 level in injured cortex and hippocampus post TBI. The results suggest a protective effect and therapeutic potential of H_2_S in the treatment of brain injury and the protective effect against TBI may be associated with regulating apoptosis and autophagy.

## Introduction

Traumatic brain injury (TBI) is a serious public health problem affecting millions of people in the world. Each year, TBI contributes to a substantial number of deaths and cases of permanent disability. TBI initiates a series of biophysiological and pathological reactions, including activation of excitatory amino acids receptor, Ca^2+^ overload, mitochondrial injury and energy metabolic blockage, production of oxyradical, caspases activation, and activation of inflammatory reaction [1], [2], that contribute to subsequent tissue damage and associated neuronal cell death, such as apoptosis, necrosis, necroptosis, and autophagy. Current standards of care in acute, subacute and chronic phases of injury are primarily supportive, however, effective pharmacological therapy remains limited [3]. Searching for neuroprotective agents, which can reduce injurious biochemical and molecular signal pathways or enhance the protective pathways, may be a promising therapeutic strategy for the treatment of TBI.

Hydrogen sulfide (H_2_S) is a colorless gas with an odour of rotten eggs that until recently was only considered to be a toxic environmental pollutant with little or no physiological significance. However, the past few years have demonstrated its role in many biological systems and it is becoming increasingly clear that H_2_S is likely to join nitric oxide (NO) and carbon monoxide (CO) as a major player in mammalian biology [4], [5]. As an almost ubiquitous bioactive molecule, H_2_S exerts important regulatory effects in several biological systems [6]. H_2_S has been found to influence heart contractile functions and may serve as a cardioprotectant for treating ischemic heart diseases and heart failure [7]. H_2_S treatment represents a novel therapeutic strategy to prevent acute lung injury induced by high tidal volume (HVT) ventilation [8]. Moreover, it is pivotally involved in the control of important functions in the central nervous system (CNS). H_2_S facilitates the induction of hippocampal long-term potentiation by enhancing the activity of N-methyl D,L-aspartate (NMDA) receptors [5]. H_2_S induces Ca^2+^ influx in astrocytes that propagates to the surrounding astrocytes as Ca^2+^ waves [9], [10]. H_2_S is also involved in CNS pathologies such as stroke and Alzheimer's disease (AD). In stroke, H_2_S appears to act as a mediator of ischemic injuries and thus inhibition of its production has been suggested to be a potential treatment approach in stroke therapy [11], [12]. It was reported that the characteristic memory deficiency in AD may be related to reduced H_2_S [13] and administration of NaHS could provide a therapeutic approach for AD [14]. Sufficient evidence has accumulated in support of H_2_S acting as a signaling molecule in the mammalian CNS. This field is still in its infancy and much will be learnt in the near future about the central roles play by H_2_S in health and disease.

Despite the substantial literature on neuroprotective effects of hydrogen sulfide in various disorders and injury, it is not known whether hydrogen sulfide can protect against TBI in mice. The lowered endogenous H_2_S level was found in the cortex and hippocampus of mice after TBI in our previous study [15]. Although the study may not be able to tell whether the lowered H_2_S in these models is a causative mechanism or just a correlative finding in the development of TBI, these interesting findings impel us to continue to study the therapeutic value of exogenous application of H_2_S. In the present study, we established a model of TBI aiming to determine whether supplementation with H_2_S would impart any tissue protective effects against brain injury and explore its potential neuroprotective mechanism through apoptotic and autophagic pathways.

## Materials and Methods

### Animals and Drug treatments

Adult male CD1 mice with an average body weight of 23 g (20 to 25 g) were used in this study. Sodium hydrosulfide (NaHS), an H_2_S donor, was obtained from Sigma (Sigma-Aldrich, St. Louis, MO) and dissolved in saline. For drug time effects assays, NaHS was intraperitoneally (i.p.) injected 30 min before or 15 min, 30 min, 1 h, 2 h, 4 h after TBI; For drug dosage effects assays, NaHS (0.1, 1, 5, 10, 25, 45µmol/kg) was i.p. injected 30 min before TBI. Animals were grouped into sham, TBI and TBI+NaHS groups. Sham-injured mice received craniotomy without TBI. All the animal procedures were approved by the Institutional Animal Use and Care Committee at Soochow University and conducted in accordance with the guidelines of Animal Use and Care of the National Institutes of Health (NIH) and the ARRIVE (Animal Research: Reporting In Vivo Experiments). All efforts were made to minimize the numbers of animals used and ensure minimal suffering. In all experiments, data were obtained by investigators blinded to study group.

### Traumatic brain injury model

The TBI model was used as previously described [15]. The CD1 mice were deeply anesthetized with chloral hydrate (4% solution) and surgery was performed under aseptic conditions and mounted in a stereotaxic system (David Kopf Instruments, Tujunga, California). The following steps were all performed using aseptic techniques. A midline incision on the scalp exposed the skull, without requiring muscle retraction. Craniotomy was performed by hand-held trephine. For the trephine method, a 5-mm diameter manual trephine (Roboz Surgical Instrument Co., Gaithersburg, MD) was carefully used to penetrate the skull for removal of the bone flap. Mice were subjected to TBI in left part of the brain (bone flap centered at the bregma+ 3.0 mm, lateral left 2.7 mm) using a weight-drop device: a 40 g weight dropped from 20 cm onto a 4 mm diameter footplate resting on the dura with a controlled depth of 1.0 mm, as described previously [16], [17]. The reproducibility and consistency of this TBI model were ensured by the accurate location, hit pressure, depth, and hitting duration. The craniotomy which did not significantly affect physiological parameters (arterial pressure, heart rate or body weight) was closed immediately after TBI [17]. For the sham operation group, only the surgical procedure was performed on animals without cortical impact. Animals were housed under a 12 h light/dark cycle in a pathogen-free area with free access to water and food. All surgical interventions and postoperative animal care were carried out in accordance with the NIH Guide for the Care and Use of Laboratory Animals and were approved by the Chinese National Committee to the Use of Experimental Animals for Medical Purposes, Jiangsu Branch. All efforts were made to minimize the number of animals used and their suffering.

### CWC measurements

For time course of TBI induced brain edema evaluation, animals were anesthetized with 4%chloral hydrate and decapitated at 1 h, 6 h, 12 h, 1d, 2d, 3d and 7d time point after TBI; For drug dosage effect and time validity evaluation, animals were anesthetized with 4%chloral hydrate and decapitated 24 h after TBI. The brains were removed and placed in a glass petri dish. CWC was measured with a drying method [18]. The cerebellar tissue was discarded, the right and left hemispheres were separated along the anatomic midline, and the wet weight of each hemisphere was measured. The tissues were completely dried in an oven at 100°C for 5 days, and the dry weight of each hemisphere was recorded. The percentage water content (% water) was calculated according to the Elliott formula for each hemisphere: 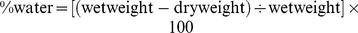 [19].

### Evaluation of Motor and Morris Water Maze Performance

Vestibulomotor function was assessed using a wire-grip test [20]. Mice were placed on a metal wire (45 cm long) suspended 45 cm above a foam pad and were allowed to traverse the wire for 60 seconds. The latency that a mouse remained on the wire within a 60 seconds interval was measured, and wire-grip scores were quantitated using a five-point scale. A score of one point was given if the mouse failed to hold on to the wire with both sets of forepaws and hind paws together; two points were given if the mice held on to the wire with both forepaws and hind paws but not the tail; three points were given if the mouse used its tail along with both forepaws and both hind paws; four points were given if the mouse moved along the wire on all four paws plus tail; and five points were given if mice that scored four points also ambulated down one of the posts used to support the wire. Mice that were unable to remain on the wire for less than 30 secs were given a score of zero. The wire-grip test was performed in triplicate and an average value calculated for each mouse on each day of testing.

The Morris water maze (MWM) task was used to evaluate spatial memory performance as described previously[20], [21]. The apparatus consisted of a circular black-colored water tank (120 cm in diameter and 50 cm high) filled with water to 29 cm depth with several highly visible cues located on the walls of each of the four quadrants. The water in the tank was colored by black non-toxic food pigment and the temperature was maintained 21°C to 25°C. A clear plexiglass goal platform 5 cm in diameter was positioned 0.5 cm below the water's surface approximately 15 cm from the southwest wall. Each mouse was subjected to a series of 4 to 8 trials per day. For each trial, mice were randomized to one of four starting locations (north, south, east, or west) and placed in the pool facing the wall. Mice were given a maximum of 60 seconds to find the submerged platform. If the mouse failed to reach the platform by the allotted time, it was placed on the platform by the experimenter and allowed to remain there for 10 seconds. Mice were placed in a warming chamber for at least 4 min between trials. To control for possible differences in visual acuity or sensorimotor function between groups, two trials were performed using a visible platform raised 0.5 cm above the surface of the water. Performance in the MWM was quantitated by latency to find the platform. To minimize potential variability in performance due to daily environmental differences, mice were always tested concomitantly in motor and MWM tasks. The time to reach the visible platform were recorded and analyzed. Trajectories and latencies of trials were monitored and achieved using a video camera and analyzed with a tracking device and software (Chromotrack 3.0, San Diego Instruments).

### Administration of Propidium Iodide and Detection of Propidium Iodide-Positive Cells

Propidium iodide (PI; 10 mg/mL; Sigma-Aldrich Corporation, St Louis, MO, USA) was diluted in 0.9% NaCl and 0.4 mg/kg was administered 1 h before killing by intraperitoneal injection in a total volume of not more than 100 µL [22], [23]. Mice were killed at 24 h after brain injury, the brains frozen in nitrogen vapor, and cryostat brain sections (12 µm) were cut at 150 to 200 µm intervals from the anterior to posterior hippocampus (bregma−1.90 to −3.00). The cryostat sections were placed on poly-L-lysine slides and stored at −80°C. All cortical regions of brain were chosen from 200× cortical fields from within contused cortex. Propidium iodide-positive cells were quantitated in cortex and hippocampus in three brain sections separated by at least 150 to 200µm [22]. For detection of PI-labeled cells, brain sections were fixed in 100% ethanol for 10 mins at room temperature, coverslipped with Permount (Biomeda, Foster City, CA, USA) and photographed on a Nikon Eclipse T300 fluorescence microscope (Tokyo, Japan) using excitation/emission filters at 568/585 nm for PI.

### Western Blot Analysis

Mice were given an overdose of chloral hydrate and sacrificed at different time points post-operatively (n = 3 for each time point), tissues from the hippocampus and cerebral cortex of the injured hemisphere surrounding the wound (extending 2 mm to the incision) were for detecting the expression of protein by western blotting technique respectively. To prepare lysates, frozen brain tissue samples were minced with eye scissors in ice. The samples were then homogenized in lysis buffer (1% NP-40, 50 mmol/L Tris, pH 7.5, 5 mmol/L EDTA, 1% SDS, 1% sodium deoxycholate, 1% Triton X-100, 1 mmol/L PMSF, 10 µg/ml aprotinin, and 1 µg/ml leupeptin) and clarified by centrifuging for 20 min in a microcentrifuge at 4°C. After determination of its protein concentration with the Bradford assay (Bio-Rad), the resulting supernatant (50 µg of protein) was subjected to SDS-polyacrylamide gel electrophoresis (PAGE). The separated proteins were transferred to a polyvinylidine difluoride membrane (Millipore) by a transfer apparatus at 350 mA for 1.5 h. The membrane was then blocked with 5% nonfat milk and incubated with primary antibody against procaspase-3 (1:500, Santa Cruz Biotechnology, Santa Cruz, CA, USA), cleaved caspase-3 (1:500; Bioword Technology, Minneapolis, MN, USA), Bcl-2 (1:1000; Bioword Technology, Minneapolis, MN, USA), Beclin-1 (1:500, Santa Cruz Biotechnology, Santa Cruz, CA, USA), Vps34 (1:300; Santa Cruz Biotechnology, Santa Cruz, CA, USA), LC3B (1:3000, Abcam, Cambridge, MA, USA), p62 (1:500, Santa Cruz Biotechnology, Santa Cruz, CA, USA) or GAPDH (1:10000; Bioword Technology, Minneapolis, MN, USA). After incubating with an anti-rabbit horseradish peroxidase-conjugated secondary antibody, protein was visualized using an enhanced chemiluminescence system (ECL, Pierce Company, USA).

### Statistics analysis

All data were expressed as mean±SEM. PI-positive cell count were analyzed by the rank-sum test. Motor and MWM test data (hidden and visible platform acquisition latencies) were analyzed by two factor repeated measures analysis of variance (ANOVA; for group and time) followed by post hoc Bonferroni's test for multiple comparisons. Data on the probe trial were analyzed using one-way ANOVA analysis followed by post hoc Tukey's test for multiple comparisons. Western blot data were carried out by one-way ANOVA with Dunnett t test. All analyses were performed with SPSS statistical package (version 13.0 for Windows, SPSS Inc., USA). For all comparisons, P<0.05 was regarded as significant.

## Results

### Hydrogen sulfide reduces TBI-induced brain edema

**Figure 1:**
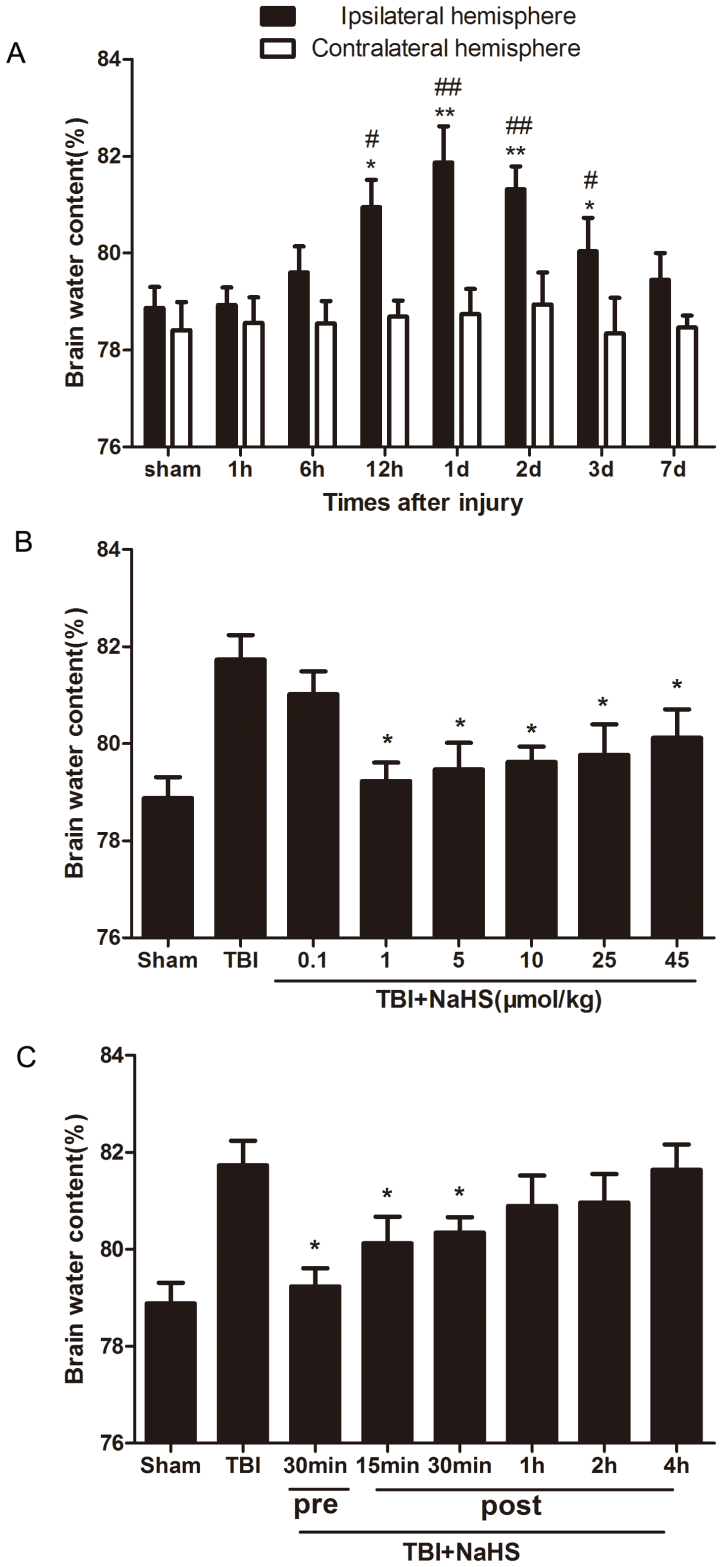
Treatment with H_2_S attenuated TBI-induced brain edema. (A) The water content of injured hemisphere and contralateral hemisphere were measured from 1 h-7d after TBI. *P<0.05, **P<0.01 vs. sham group.^ #^P<0.05, ^##^P<0.01 vs. contralateral hemisphere group at the same time point. (B) Pretreatmented H_2_S at 30 min before TBI with different dosage from 0.1–45µmol/kg, and the brain water content were measured at 1d after TBI. *P<0.05 vs. TBI group. (C) Injected H_2_S (1µmol/kg,) at 30 minutes before or 15 min, 30 min, 1 h, 2 h, 4 h post TBI, and the brain water content were detected at 1d after TBI. *P<0.05 vs. TBI group. Data were expressed as mean ± SEM (n  = 6).

TBI led to a significant increase in the percentage of water content in the injured ipsilateral cortex (Figure 1). Compared with sham group and contralateral hemisphere, the amount of water content of the injured hemisphere increased 6 h, peaked 1d to 2d, and lasted to 3d after TBI. Pretreatment 30 min before TBI by NaHS with dosage of 1µmol/kg, 5µmol/kg, 10µmol/kg, 25µmol/kg or 45µmol/kg can reduced the percentage of water content in the injured ipsilateral cortex, but 0.1µmol/kg dosage did not have the same function. Injection of NaHS (1µmol/kg) 30 min before 30 min or 15 min, 30 min after TBI attenuated TBI induced brain edema. The result indicates that exogenous H_2_S treatment can ameliorate the development of TBI-induced brain edema.

### Hydrogen sulfide ameliorates motor deficits and improves spatial memory acquisition after TBI

**Figure 2:**
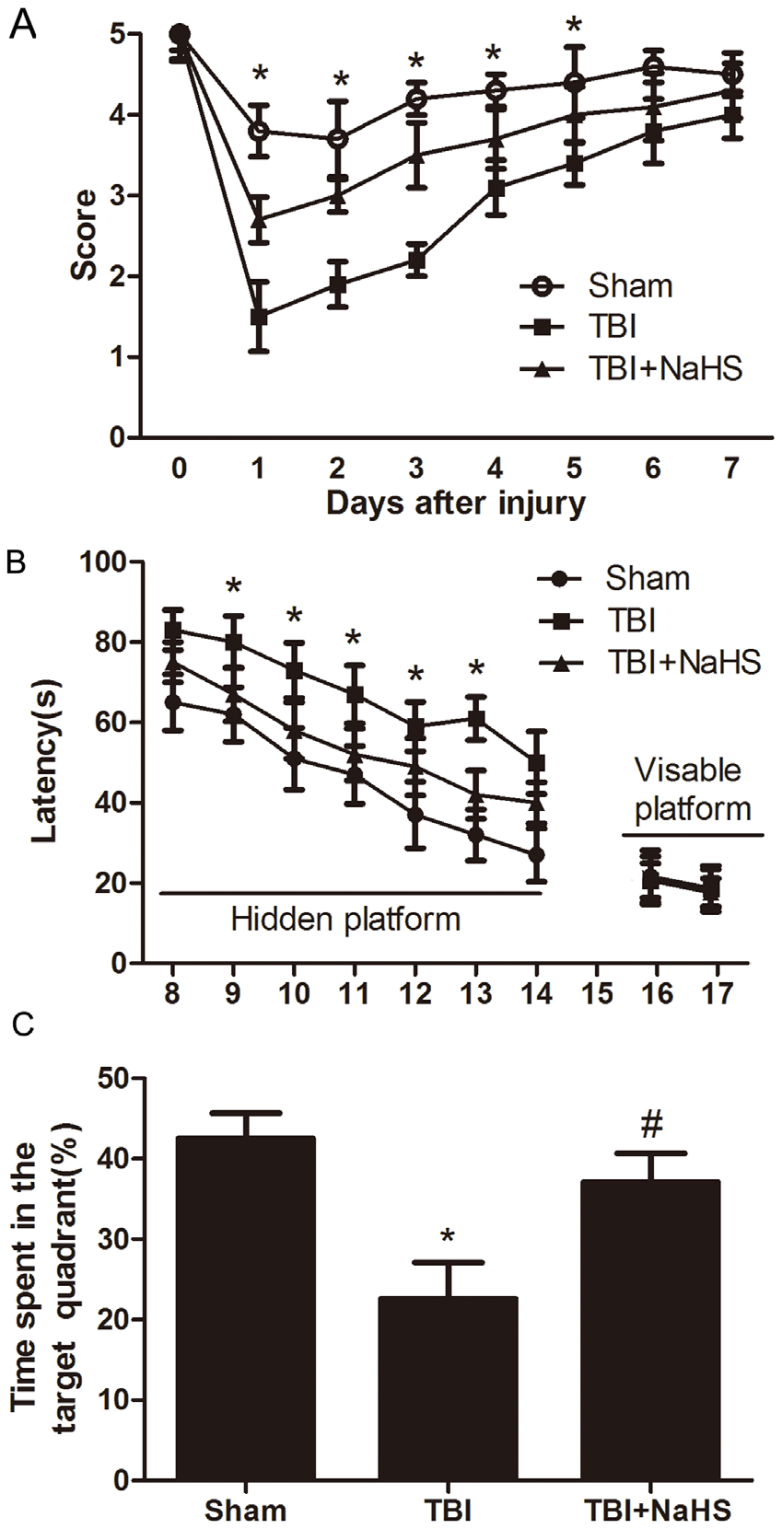
H_2_S improved the recover of TBI-induced motor deficits and improved spatial memory acquisition. (A) Motor function was assessed by a wire grip test. No difference in baseline motor function before TBI was observed between groups of mice administered H_2_S. Pretreatment with H_2_S speeded up the recover of motor function deficits in mice compared to TBI group (* P<0.05 vs. TBI group, n = 10/group). (B) Mice in TBI group had significantly longer escape latency compared with sham group in the hidden platform task; however, TBI+NaHS group had significantly lower escape latency compared with TBI group. There was no significant difference in escape latency in all three groups in the visible platform task. (C) Duration of time mice remained in the target quadrant during the probe trial of the MWM. During the probe trial, mice in TBI+NaHS showed a significant difference in distance traveled in the target quadrant compared to TBI group. (* P<0.05, vs. sham group; # P<0.05 vs. TBI group, n = 10/group).

To determine whether H_2_S treatment was associated with improved neurological outcome, we sought to perform behavior experiments. No difference in baseline motor function before TBI was observed between groups of mice. TBI elicited a significant decline in motor performance on days 1–5, which returned to basal levels on days 6 post-injury. Following TBI, pretreatment with hydrogen sulfide (1µmol/kg) improved the recovery of motor functional outcome on days 1 to 5 post TBI compared to TBI group (Figure 2).

When there were no differences in motor function between groups, morris-water maze performance was tested on days 8–17. In Morris water maze experiments, mice showed normal acquisition curves on both hidden and visible platforms, and selective quadrant search on the probe trial before suffering TBI (Figure 2). All TBI-treated animals displayed increased latencies in the ability to find the hidden platform, versus the sham group on days 8–14. After injury, animals subjected to H_2_S pretreatment demonstrated a significant decrease in the latencies, relative to TBI mice on days 9–13, thereby indicating H_2_S treatment could result in cognitive functional recovery in the TBI model. To exclude differences in visual acuity between groups during Morris-water maze testing, visible platform testing was performed on days 16 and 17. On days 16 and 17, there were no differences in latencies to find the visible platform between groups. In the probe trial of the Morris water maze test, TBI group had a significant effect on the time in target quadrant compared with sham group. Compared with TBI group, TBI+NaHS group displayed more time swimming in the target quadrant.

### Hydrogen sulfide reduces acute plasmalemma permeability in injured cells after brain injury

**Figure 3:**
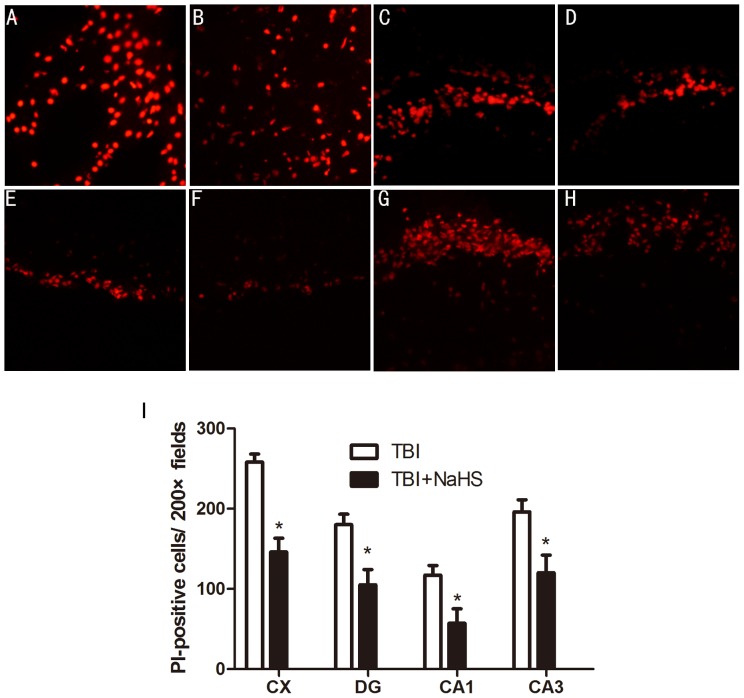
Pretreatment with H_2_S reduces PI-positive cells at 6 h after TBI in the injured cortex and hippocampus. Representative photomicrographs showed that pretreatment with H_2_S reduced numbers of PI-positive cells in the cortical (A and B), dentate gyrus (C and D), CA1 (E and F), CA3 (G and H) brain regions after brain injury in TBI+NaHS group (B, D, F, H) compared to TBI group (A, C, E, G). Original magnification × 200. I, quantitation of PI-positive cells in the injured cortex, dentate gyrus, CA1 and CA3 regions. *P<0.05 versus TBI group (n = 10 per group).

Plasmalemma permeability is a hallmark of apoptotic and autophagic, including necrotic cell death. To begin to address cellular mechanisms of reduced postinjury tissue damage, we assessed the effect of H_2_S treatment on loss of plasmalemma integrity in the cortical and hippocampal brain regions using in vivo (Figure 3). Compared with TBI group, mice administered H_2_S before TBI had decreased numbers of PI-positive cells at 6 h after TBI in the injured cortex, dentate gyrus, CA1 and CA3 regions. In contrast to injured mice, PI-positive cells were not detected in brain regions from sham group, or in the contralateral hemisphere of injured mice.

### Hydrogen sulfide reverses TBI-induced caspase-3 cleavage and Bcl-2 decline

**Figure 4:**
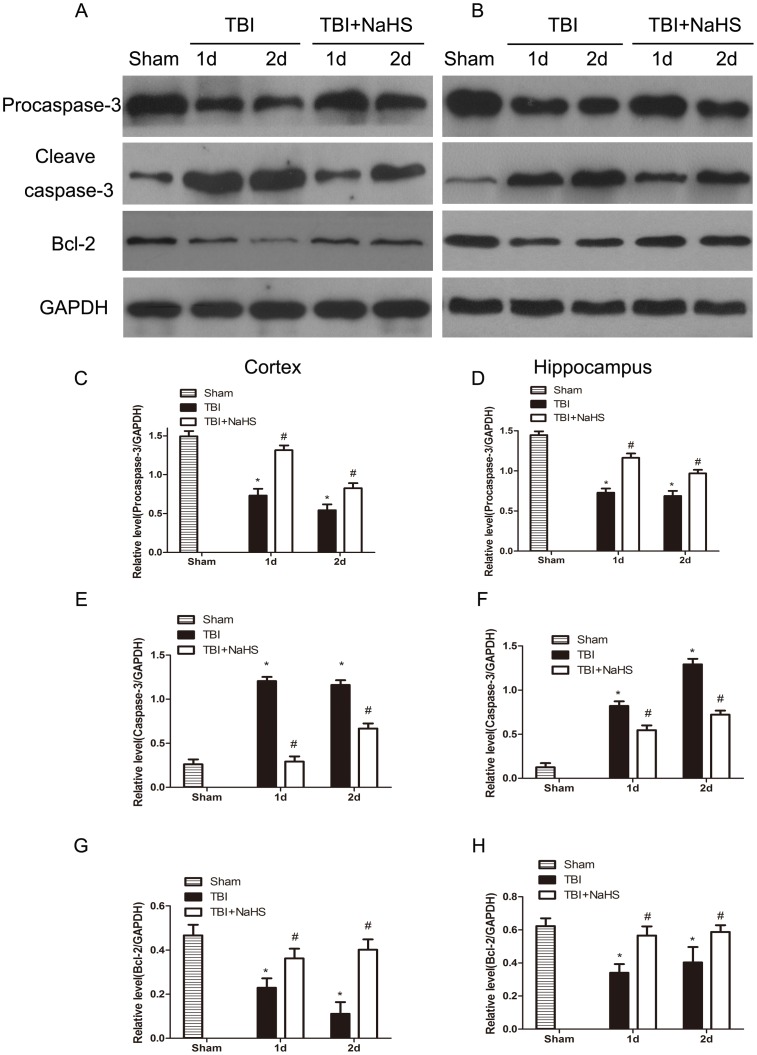
TBI-induced cleavage of caspase-3 and Bcl-2 decline were reversed by H_2_S pretreatment. Sample immunoblots probed for caspase-3 (procaspase-3 and cleaved caspase-3), Bcl-2 and GAPDH are showed above. The *bar chart* below demonstrates caspase-3 (procaspase-3 and cleaved caspase-3) and Bcl-2 relative to GAPDH. TBI-induced up-regulation of cleaved caspase-3 and down-regulation of Bcl-2 were inhibited by H_2_S in the cortex (A) and hippocampus (B). Optical densities of the protein bands were quantitatively analyzed with Sigma Scan Pro 5 and normalized with loading control GAPDH. Semiquantitative analysis (relative optical density) of the intensity of staining of procaspase-3 to GAPDH in the cortex (C) and hippocampus (D). Semiquantitative analysis (relative optical density) of the intensity of staining of cleaved caspase-3 to GAPDH in the cortex (E) and hippocampus (F). Semiquantitative analysis (relative optical density) of the intensity of staining of Bcl-2 to GAPDH in the cortex (G) and hippocampus (H). The data are means ± SEM (n = 3, *P<0.05, TBI group vs. sham group; # P<0.05 TBI+NaHS group vs. TBI group at the same time point).

Because plasmalemma permeability may also be associated with apoptotic mechanisms, we next assessed the effect of H_2_S on caspase-3 activation in the same brain regions used to assess procaspase-3, cleaved caspase-3 and Bcl-2 expression at 1d and 2d after TBI (Figure 4). Western blot results demonstrated that TBI leads to an decrease in procaspase-3 levels after injury. The protein levels of cleaved caspase-3 had a marked increase at day 1 and 2 after TBI, and the protein levels of Bcl-2 had a modestly reduction at day 1 and 2 after TBI in the injured cortex and hippocampus. We demonstrated a decrease in procaspase-3 at day 1 and 2 following TBI, consistent with an increased activation of caspase-3. H_2_S pretreatment significantly reversed the effects on cleaved caspase-3 increase and Bcl-2 decrease in the injured cortex and hippocampus after TBI, demonstrating H_2_S suppressed TBI induced cell apoptosis.

### Hydrogen sulfide inhibits TBI-induced autophagic activation

**Figure 5:**
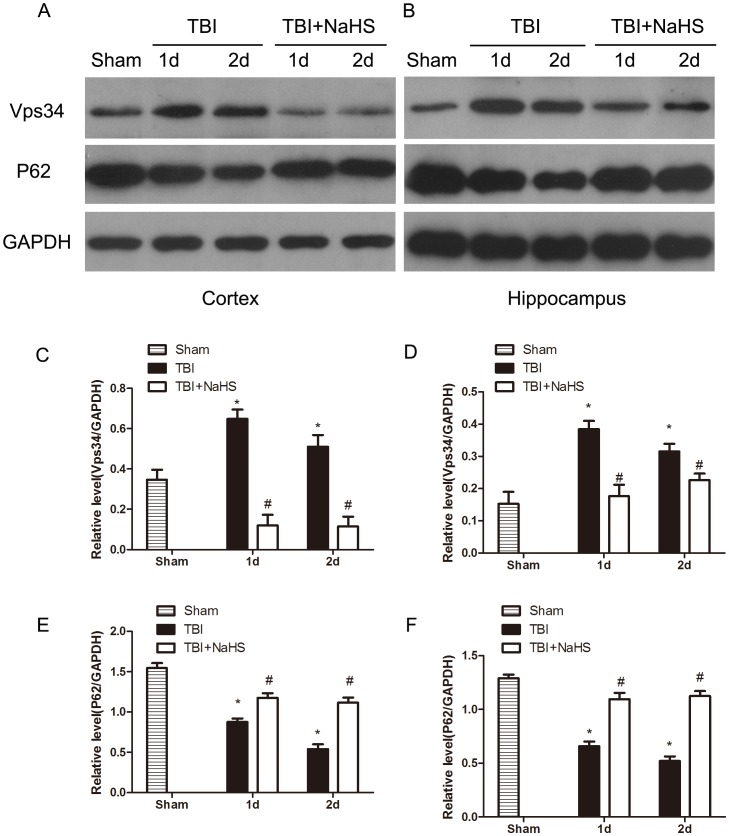
H_2_S pretreatment suppressed TBI-induced the increase of Vps34 and P62. Sample immunoblots probed for Vps34, P62 and GAPDH are showed above. The *bar chart* below demonstrates the ratio of Vps34 and P62 relative to GAPDH. TBI-induced up-regulation of Vps34 and down-regulation of P62 were inhibited by H_2_S in the cortex (A) and hippocampus (B). Optical densities of the protein bands were quantitatively analyzed with Sigma Scan Pro 5 and normalized with loading control GAPDH. Semiquantitative analysis (relative optical density) of the intensity of staining of Vps34 to GAPDH in the cortex (C) and hippocampus (D). Semiquantitative analysis (relative optical density) of the intensity of staining of P62 to GAPDH in the cortex (E) and hippocampus (F). The data are means ± SEM (n = 3, *P<0.05, TBI group vs. sham group; # P<0.05 TBI+NaHS group vs. TBI group at the same time point).

**Figure 6:**
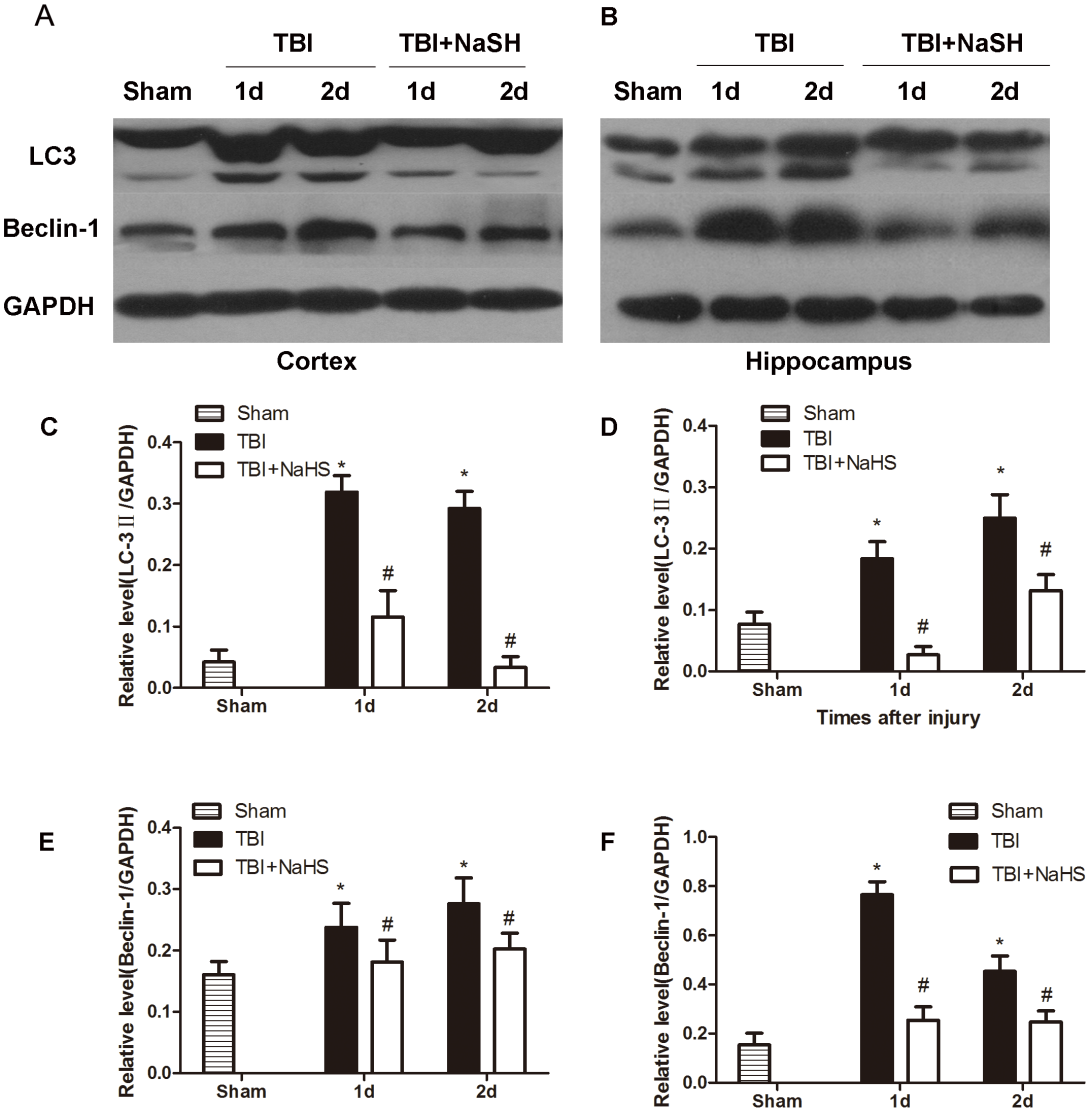
H_2_S pretreatment reversed TBI-induced the LC3II and Beclin-1 increase. Sample immunoblots probed for LC3II, Beclin-1 and GAPDH are showed above. The *bar chart* below demonstrates the ratio of LC3II and Beclin-1 relative to GAPDH. TBI-induced up-regulation of LC3II and Beclin-1 was inhibited by H_2_S in the cortex (A) and hippocampus (B). Optical densities of the protein bands were quantitatively analyzed with Sigma Scan Pro 5 and normalized with loading control GAPDH. Semiquantitative analysis (relative optical density) of the intensity of staining of cleaved LC3II to GAPDH in the cortex (C) and hippocampus (D). Semiquantitative analysis (relative optical density) of the intensity of staining of Beclin-1 to GAPDH in the cortex (E) and hippocampus (F). The data are means ± SEM (n = 3, *P<0.05, TBI group vs. sham group; # P<0.05 TBI+NaHS group vs. TBI group at the same time point).

**Figure 7:**
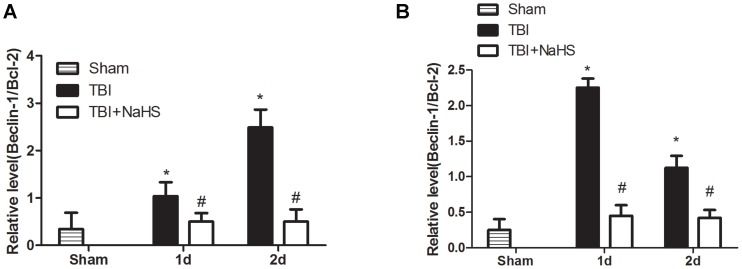
H_2_S pretreatment suppressed TBI-induced the increase of the Beclin-1/Bcl-2 ratio. H_2_S pretreatment inhibited TBI-induced the increase of the Beclin-1/Bcl-2 ratio in the cortex (A) and hippocampus (B). The data are means ± SEM (n = 3, *P<0.05, TBI group vs. sham group; # P<0.05 TBI+NaHS group vs. TBI group at the same time point).

To investigate whether the neuroprotective effect of hydrogen sulfide pretreatment on TBI was directly bound up with autophagic activity, the protein levels of Vps34, p62, Beclin-1 and LC3II were determined (Figure 5, 6). Formation of LC3II interacted with the elongation and maturity of autophagosome formation and the expression of LC3II protein could reflect autophagic activity. The LC3II expression was markedly increased post TBI, and pretreatment with H_2_S significantly decreased LC3II expression in the injured cortex and hippocampus. Recently, p62 protein has been suggested to correlate with ubiquitinated proteins and LC3, which may regulate the selective autophagic clearance of protein aggregates [24]. After TBI, the decreased protein level of p62 was detected in the injured cortex and hippocampus. We found that H_2_S pretreatment maintained the p62 protein level, versus the TBI group. Beclin-1 was a key protein which has been involved in the regulation of autophagy [25]. Pretreatment with H_2_S resulted in a marked decrease in Beclin-1 protein level at day 1 and 2 post TBI, versus TBI group. In addition, Vps34 protein has been suggested to interact with Beclin-1 and other autophagic related proteins, which may regulate the origination of autophagosome formation [26]. The protein level of Vps34 was immediately increased post TBI, and pretreatment with hydrogen sulfide significantly decreased the relative protein level of Vps34 in the injured cortex and hippocampus post TBI. Furthermore, the Beclin-1/Bcl-2 ratio has previously been demonstrated to be a significant and more sensitive measurement of the regulation of autophagy [27], [28]. Pretreatment with hydrogen sulfide significantly decreased the Beclin-1/Bcl-2 ratio in the injured cortex and hippocampus post TBI in this study (Figure 7).

## Discussion

TBI is considered as a major health problem that is a frequent cause of death and disability and makes considerable demands on health services. There was limited advance in the therapeutic strategies to counter brain injury. Except for conservative management, neuroprotection and neurorecovery are still the main therapeutic strategies under development. An ideal neuroprotectant would be non-toxic, easily administered, permeable at the blood-brain barrier (BBB), and offer protection at all stages of injury, including prophylaxis. H_2_S has shown some of these properties [29]. In this study, our data demonstrated that H_2_S treatment reduced brain edema, improved the recovery of motor and cognitive dysfunction and suppressed cellular plasmalemma integrality disruption in a mouse model of TBI. Administration of H_2_S also showed H_2_S had an anti-apoptotic activity and down-regulated the expression of autophagy-related proteins including Vps34, Beclin-1 and LC3II after brain injury.

Cerebral edema has been reported to be one of the major factors leading to the high mortality and morbidity associated with patients with TBI [30]. A recent study interestingly showed that H_2_S at a low concentration significantly attenuated the injury in a mild focal cerebral ischemia rat model [31], [32]. The time profile of brain edema after TBI was determined, and dosage and time effects of H_2_S were detected in this study. We observed that pre-treatment 30 min before TBI by NaHS with dosage of 1 µmol/kg, 5 µmol/kg, 10 µmol/kg, 25 µmol/kg or 45 µmol/kg can suppress TBI induced brain edema, but 0.1 µmol/kg dosage did not have the same function. Our study showed that lower (1 µmol/kg) dose of NaHS had more significant protective effect on the brain edema than higher dose (45µmol/kg) of NaHS. Consistent with our results, in vitro and in vivo studies demonstrate that low concentrations of H_2_S is neuroprotective, whereas higher concentrations of H_2_S have been shown to magnify cerebral damage [12], [32]. Since the best therapeutic effect of NaHS was found when given 30 min prior to TBI, we continued with this study setup to explore its effect on brain function in mice after injury.

To investigate whether H_2_S can ameliorate motor and cognitive dysfunction after TBI, motor test and Morris water maze were performed. We found in the present study that systemic treatment with H_2_S significantly ameliorated traumatic-induced motor and resulted in a significant amelioration in injured mice in hidden platform and probe trials (the latter being a sensitive and specific test of spatial learning) in this study. It is likely that motor deficits do not significantly contribute to MWM deficits in TBI mice, because visible platform latencies (which control for differences in postinjury motor function) were not different between sham-injured and TBI animals. The finding that H_2_S-treated and sham groups had similar performance in visible platform tests suggested that improved MWM performance was not caused by differences in motivation, swim speed, or other nonspatial brain functions, but likely represents improvement of spatial memory acquisition and retention. In fact, previous studies demonstrated that, following TBI, hippocampal cell death is believed to be associated with the cognitive impairment observed in both clinical and experimental settings [33], [34]. Therefore, besides the improving cellular resiliency and neural plasticity mechanism, reducing TBI-induced neural cell lost may be also contributed to the neuroprotective effects of H_2_S.

Using in vivo PI labeling to follow the fate of cells injured by TBI, Whalen et al. found that plasmalemma damage occurs early and before the onset of DNA fragmentation in brain regions vulnerable to traumatic cell death and portend a fatal outcome in cells injured early after TBI (Whalen et al. 2008b). Our aforetime study showed that the number of PI-positive cells increased at 6 h, reached a peak on the second day, then declined [16]. To reduce or even prevent damage in its earliest stages, the number of PI-positive cells was counted at 6 h in this study. Compared to TBI group, H_2_S pretreatment could decrease the number of PI-positive cells in injured cortex, dentate gyrus, CA1 and CA3 regions. These results were consistent with our aforetime results which demonstrated that administration of H_2_S significantly reduced brain lesion volume and neuronal injury in the CA1 hippocampus [15]. Our PI labeling data further reveal that H_2_S may protect brain from neuronal loss in both cortex and hippocampus. These findings indicate that H_2_S may be a potential therapy for TBI.

Studies show that secondary cell death may eventually account for up to 40% of the total tissue loss, thus playing a determining role in the outcome following TBI and hence presenting an important drug target for neuroprotective treatment(Zweckberger et al. 2003, Plesnila et al. 2007). Cell death is broadly classified into three types: necrosis, apoptosis (type 1 PCD) and autophagy (type 2 PCD) [35]. In contrast to necrosis, PCD is a highly regulated and energy demanding process and may be initiated by the primary necrosis [36]–[38]. There were no specific methods to detect necrosis, thus we focused on apoptosis and autophagy. Apoptosis plays a significant role in the pathophysiology of brain injury in TBI model [2], [39]. Among these genes, Bcl-2 and Caspase-3 are widely regarded as the most important apoptotic regulators, and their relative levels determine the fate of cells. We found that NaHS treatment resulted in decreased cleaved caspase-3 protein levels but increased the Bcl-2 protein level in the cortex and hippocampus. These data suggested that H_2_S inhibited the progression of apoptosis in the cortex and hippocampus after brain injury by TBI.

Increased autophagy is observed in multiple and distinct experimental models of brain injury including trauma [40]. However, it is not known whether the role of autophagy is protective or detrimental for neural tissue after brain injury. It is possible that autophagy's role after brain injury is dependent upon the cell's capacity to respond in relation to the cumulative burden of damaged or dysfunctional macromolecules and organelles. If the increase in autophagic capacity is insufficient, augmenting autophagy would likely be beneficial. The increase in autophagic capacity is in excess, and inhibiting autophagy may be beneficial. Thus, the role of autophagy may be dictated by whether or not it can meet intracellular demands. TBI results in damage to proteins, lipids, and organelles secondary to activation of proteases and lipases, free radical damage, and a host of other mechanisms[41]. Oxidative stress contributes to overall neuropathology after TBI, at least in part by initiating or influencing autophagy[42]. A logical and effective therapeutic option is to reduce the autophagic burden after brain injury (e.g., using antioxidants) [40]. H_2_S, as a potent antioxidant, can protect human cultured neuron cells from oxidative stress, and this effect opens up a promising perspective for the possible role of H_2_S in protection from brain injury [4], [43]. We found that pretreatment with H_2_S observably inhibited TBI-inducted elevation of the LC3II and Beclin-1, and maintained p62 levels in the injured cortex and hippocampus post TBI, suggesting a decreased ability of autophagy to degrade endogenous substrates. Growing evidence suggests that autophagy contributes to neuronal cell death in TBI. Including our previous work, studies showed that autophagy was increased post brain injury and inhibition of autophagy by 3-MA improves functional outcome after traumatic brain injury in mice [17]. Suppressing autophagy may be another protective mechanism of H_2_S in our mouse TBI model, but the possible mechanism of suppressing-autophagy by H_2_S should be exploded in our TBI model.

Although ample evidence demonstrated enhanced autophagy in neuronal death following TBI, the signaling pathways regulating its activation remain poorly defined. Beclin 1, the mammalian orthologue of yeast Atg6, has a central role in autophagy, which interacts with several cofactors to regulate the lipid kinase Vps-34 (Vacuolar sorting protein 34) and promote formation of Beclin 1-Vps34-Vps15 core complexes, thereby inducing autophagy [44]. So we detected Vps34 protein levels in this study and the result indicated that H_2_S pretreatment prohibited the increase of Vps34 protein in the injured cortex and hippocampus. One of the key mechanisms for control of autophagy is the modulation of the interaction between the autophagic protein Beclin 1 and the members of the anti-apoptotic Bcl-2 family [45]. Beclin 1 was also identified as a Bcl-2-interacting protein through its BH3 domain [46]. The binding of Bcl-2 to Beclin 1 disrupts the association of Beclin 1 with PI3K, Vps34 and p150, therefore inhibiting autophagy [27]. The Bcl-2/Beclin 1 interaction is a very important checkpoint in the regulation of autophagy, and previous study proved that the Beclin-1/Bcl-2 ratio increased in the injured cortex in a rat CCI model [28]. In this study, H_2_S pretreatment can prohibit the increase of the Beclin-1/Bcl-2 ratio in injured cortex and hippocampus after TBI which indicated that H_2_S can suppress apoptosis and autopahgy by regulating Beclin-1-Vps34 interaction.

In conclusion, the present study demonstrates that systemic administration of H_2_S ameliorates brain edema and behavioral symptoms in TBI models. H_2_S may serve as a neuroprotectant to treat TBI-induced brain injury via anti-apoptosis and suppression of excessive activation of autophagy therefore has potential clinical therapeutic value for treatment of TBI.
